# Integrating clinical and public health knowledge in support of joint medical practice

**DOI:** 10.1186/s12913-020-05886-z

**Published:** 2020-12-09

**Authors:** Jean-Pierre Unger, Ingrid Morales, Pierre De Paepe, Michel Roland

**Affiliations:** 1 Department of Public Health, Institute of Tropical Medicine, Nationalestraat 155, B-2000 Antwerp, Belgium; 2 Office de la Naissance et de l’Enfance, French Community of Belgium, Chaussée de Charleroi 95, B-1060 Brussels, Belgium; 3 Département de Médecine Générale, Université Libre de Bruxelles, Route de Lennik, 808, BP 612/1, B-1070 Brussels, Belgium

**Keywords:** Medical and public health practice, Health epistemology, Medical education, Medical research, Health management, Health policy

## Abstract

**Background:**

Strong relations between medicine and public health have long been advocated. Today, professional medical practice assumes joint clinical/public health objectives:

- GPs are expected to practice community medicine;
- Hospital specialists can be involved in disease control and health service organisation;
- Doctors can teach, coach, evaluate, and coordinate care;
- Clinicians should interpret protocols with reference to clinical epidemiology.
- Public health physicians should tailor preventive medicine to individual health risks.

This paper is targeted at those practitioners and academics responsible for their teams’ professionalism and the accessibility of care, where the authors argue in favour of the epistemological integration of clinical medicine and public health.

**Main text:**

Based on empirical evidence the authors revisit the epistemological border of clinical and public health knowledge to support joint practice. From action-research and cognitive psychology, we derive clinical/public health knowledge categories that require different transmission and discovery techniques.

The knowledge needed to support the universal human right to access professional care bridges both clinical and public health concepts, and summons professional ethics to validate medical decisions. To provide a rational framework for teaching and research, we propose the following categories:

- ‘Know-how/practice techniques’, corresponding a.o. to behavioural, communication, and manual skills;
- ‘Procedural knowledge’ to choose and apply procedures that meet explicit quality criteria;
- ‘Practical knowledge’ to design new procedures and inform the design of established procedures in new contexts; and
- Theoretical knowledge teaches the reasoning and theory of knowledge and the laws of existence and functioning of reality to validate clinical and public health procedures.

Even though medical interventions benefit from science, they are, in essence, professional: science cannot standardise eco-biopsychosocial decisions; doctor-patient negotiations; emotional intelligence; manual and behavioural skills; and resolution of ethical conflicts.

**Conclusion:**

Because the quality of care utilises the professionals’ skill-base but is also affected by their intangible motivations, health systems should individually tailor continuing medical education and treat collective knowledge management as a priority. Teamwork and coaching by those with more experience provide such opportunities. In the future, physicians and health professionals could jointly develop clinical/public health integrated knowledge. To this end, governments should make provision to finance non-clinical activities.

## Background

Closer ties between clinical medicine and public health have been advocated more recently [1, 2] as well as in the past. Writing for the practitioners and academics who feel themselves responsible for teamwork and professionalism in their services and also for accessibility of care in the community, we argue for the effective epistemological integration of these two disciplines.

This article explores the empirical rationale for, and ways to integrating clinical and public health knowledge, using an analysis of existing practitioner expertise meant to facilitate universal access to medical care and ethical practice.

The social relevance of medical systems is becoming a worldwide concern. The 2017 World Summit on Social Accountability, for example, strove to redefine the pathway of social accountability for the education of health professionals in the future (https://thenetworktufh.org/2017conference/). A charter developed as the main product of the (clinical) Medical Professionalism Project, identified a commitment to the primacy of the individual patient’s welfare, social justice (and hence the fair distribution of health care resources) as core principles that should underpin physicians’ professionalism [3].

From the charter’s perspective, health care providers should not set limits to their concerns for the health care needs of individual patients. In addition, they should tackle social justice and apply social determinants of health and public health problems to ensure that, not only their patients, but also all individuals can access good quality health care [4]. They can incorporate this approach into their practice, by striving to deliver equal care to all patients, whatever their incomes [5] and also by performing advocacy [6].

Public health science, for its part, has been defined as accumulated knowledge about collective health protection. According to WHO, “Public Health is defined as “the art and science of preventing disease, prolonging life and promoting health through the organized efforts of society” (Acheson, 1988; WHO). Activities to strengthen public health capacities and service aim to provide conditions under which people can maintain to be healthy, improve their health and wellbeing, or prevent the deterioration of their health. Public health focuses on the entire spectrum of health and wellbeing, not only the eradication of particular diseases” [7].

Due to this focused concern on threats to health based on population risk analysis, public health scientists and international cooperation agents have, all too often, treated clinical medicine and allied professions merely as the means of controlling epidemics and population health risks, not as methods of delivering individual health care [8].

In borrowing concepts from general management sciences, public health science often fails to recognise the unique character of health systems and service delivery carried out by physicians and health professionals. In general public health science has neglected the delivery of individual healthcare, health provider-user interactions and issues with medical professionalism because of its quantitative, probabilistic methodology bias.

Explanations for epistemological stagnation underpinning the relations between public health and clinical sciences are to be found in history and in the immutable influence of social structures on health systems. As a rule, public health interventions were designed for the poor and medical care for the rich. At the end of the nineteenth century, public health officers in England were in charge of the prevention and early detection of, for example, cholera, tuberculosis, and scabies, but the government did not provide health care for the poor [9]. That was a responsibility delegated to a small number of charity hospitals.

This reality remains extant in huge areas of Africa, Latin America, and Asia. Since few doctors voluntarily choose to work in deprived neighbourhoods (except for those affiliated with certain denominational hospitals), health care for the poor is largely treated as collective public health care programme, i.e. treated solely as an aspect of public and/or private central planning.

This history still defines the epistemological borders of public health and clinical sciences. In the early 2000s, the WHO officially endorsed Health Systems Research (HSR) as a key component of public health without explicit reference to individual health care delivery [10]. Ten years later, HSR was still being portrayed as a key element of health policy and health systems without putting individual health care delivery at its core, merely mentioning it in a long list of other study topics [11]. Implementation research continued to focus on disease control programmes and biomedical interventions, unwittingly strengthening the rise of ‘inequality by disease’.

This legacy would not hamper the advancement of health care if the division between collective and individual health sciences were found to be desirable. Since a population’s well-being can only ever be as good as that of its individual members this is not the case and it is illogical to separate them.

Physicians can maximise their impact on collective health and still deliver highly individualised health care in line with eco-biopsychosocial and patient-centred care standards. To do so, they must strive to constantly improve their own professionalism and that of their environment. Alongside clinical responsibilities, doctors can reflect on their practice, build and lead teamwork, coach, educate, train, improve the organisation of their health services, coordinate and evaluate health care, contribute to disease and health risk control, undertake operational research, and lobby on health policy design. When physicians develop and connect these activities they may be called “manager physicians” even without being officially appointed, since managers are traditionally defined as persons entrusted with decision-making aimed at achieving their institutions’ predetermined goals most efficiently.

Any doctor, the authors contend, should adopt the role of ‘manager physician’ in order to use her/his knowledge and experience fully, because non-clinical activities potentiate clinical ones. For ethical reasons discussed elsewhere, [12] physicians should volunteer to do this independently of or prior to the adoption of their institutions’ policies, that is, without necessarily being instructed to do so.

We propose to call medical ethics that are based on the values of ‘non-maleficence, beneficence, autonomy, and justice…(the reference tetrad par excellence that physicians and ethicists use to resolve ethical dilemmas’ [13]) neo- Hippocratic medical ethics. The neo prefix is justified by the addition of a distributive dimension to traditional Hippocratic ethics.

Medicine, health care management and government policy ought to contribute to the human right to care – the right to access professionally-delivered individual and collective health care in universal health systems.

**Table:** 

Universal health systems can be defined as offering the full array of health care services, from community health centers and self-employed GPs to university teaching hospitals made accessible to those in need over a territory. The antonym is often called ‘segmented health systems’.	

Therefore, clinicians concerns shouldn’t merely be quality of care but also care accessibility; medical ethics; prevention and health promotion; and the management of population risks, diseases and health services. Actually, this is what all good clinicians do, bucking the trend for growing specialisation in the health sector. Similarly, public health physicians should focus on the accessibility and quality of individual care, alongside disease control.

This paper uses neo-Hippocratic’ medical practice as an *illustration* of joint clinical/public health practice. It is one illustration: Chinese acupuncturists or business-minded physicians will certainly have other views on individual/collective health practices and the relevant knowledge required.

## Main text

Based on the professional experience, totalling 150 years, of three public health physicians and that of a general practitioner each of whom have combined practical and academic background, we (the authors) justify the epistemological integration of clinical and public health medicine while discussing the necessity for social / professional ethics and the need for hybrid, clinical / public health decisions and action in medical practice. After exploring the hybrid nature of key medical responsibilities, we examine the related knowledge and determine how joint practice should be delivered, managed, and researched professionally, i.e., by people with particular skills and qualifications, sufficient autonomy, and abiding by professional ethics [14]. We revisit the epistemological border of clinical and public health knowledge to support joint practice. From action-research and cognitive psychology, we derive medical knowledge categories that require different transmission and discovery techniques, to support professionally- and socially-oriented medical practice and management. We analyse the difference between proposed neo-Hippocratic medicine and management, and the industrial, ‘generic’ management applied to healthcare. The essay is supported by a Pubmed bibliographic search with the terms ‘physicians public health knowledge needs’ (2000–2020).

How should medical (and health) professions evolve to encompass cross-responsibilities of clinicians and public health practitioners in order to improve the quality and accessibility of individual care and disease control?

### Public health activities in clinical practice

Consider first-line clinicians, general practitioners (GPs), or paediatricians. They have practical and conceptual public health responsibilities, as follows:

- In principle, physicians should combine (possibly programmed) primary, secondary, and tertiary prevention, curative care, and health promotion and tailor this mixture to the individual, family, and/or community.
- The family physician should practice community medicine – for example, to manage drug addiction or violence at home.
- To the extent that GPs must practice community medicine and deliver preventive care, they should accept responsibility for the whole community or geographic areas (territories) healthcare needs.
- Doctors should know how to avoid ‘patient’s delay’ (for example in women with a placenta praevia) and ‘doctor’s delay’ (for example in detecting TB); reduce transmission (of HIV, for example); secure continuity of care for patients; and provide longitudinal care for each type of disease.
- Good practice requires adjusting clinical protocols to local epidemiology because human resources, medical technology, or pharmaceuticals may not be available; and because disease frequency may vary significantly from neighbourhood to neighbourhood and therefore also the predictive value of signs, symptoms and test results.
- Medical practice requires the ability to read and interpret scientific, medical and policy evidence critically [15] and to do so not only from the standpoint of clinical epidemiology, [16] but also in the light of sociological and political economy concepts.
- Physicians should improve the way their health care services are organised (e.g. with regard to access to care, knowledge management, clinical coordination, reflection, doctors’ intangible motivation and health information) and shape the organisation in a way that is conducive to quality care. For example, GPs can pre-arrange communication channels with specialists to share decisions based on the patient’s health and family circumstances.

Although this list is not exhaustive, the clinical responsibilities of general practitioners not only demand their familiarity with public health concepts such as (clinical) epidemiology, disease control and health management but also the integration of clinical and public health knowledge. The correct care of the patient supposes not only curative but also preventive activities, and action on his/her environment. This similarly applies to the public health knowledge of hospital specialists because, they should also be able to deliver bio-psychosocial care, participate in the way that the health system is organised, and be involved in the organised control of diseases (e.g. nosocomial infections) and in the adjustment of patients’ average length of stay (in dialogue with their patient’s GP).

### Individual care delivery in public health programmes

Public health practitioners, for their part, should include elements of individual health care delivery in their collective health practices and mobilise clinical knowledge out of concerns for community health, in order to improve disease control programmes:

- Preventive care and health education should be tailored to individual biopsychosocial risks and demands for care, and public health interventions cannot simply be treated as a mass activity. Well-baby, antenatal, geriatric and HIV/AIDS clinics are examples of this. While risks and sometimes pathologies are multiple, the goal-oriented approach of medical practice encourages and assists individuals to achieve their maximal health potential in line with individually defined goals [17]. This approach should thus drive public health doctors to shift from chronic disease management to participatory patient management [18].
- Effective prevention requires proper treatment of inter-current episodes of illness, with consequences for the accessibility of medical care by high-risk patients and subsequently for clinical coordination.
- Disease control programmes are best carried out if epidemiologists can interpret the paradoxical results of (nosocomial, HIV, measles, etc.) surveillance and are able to give sound clinical advice and sustain a dialogue with clinicians (which is why physicians make good epidemiologists).
- Disease control programmes may crowd out individual care, with, for example, a ‘one size fits-all’ approach and a bureaucratic load that strains the quality of care [8]. This produces a Catch 22 situation in which disease control is undermined when, as is frequently the case, the relevant (diabetes, malaria, tuberculosis, etc.) control programme relies on appropriate clinical activity in first-line services to deliver its interventions [19]. There is increasing evidence that accessible, good, quality community-based clinical care provides the necessary confidence for public health interventions to be acceptable to the population. Public health programmes need to be organised in such a way that protects, rather than undermines, access to and the quality of, individual health care and clinical practice.

These responsibilities justify the need for public health physicians to be well informed about topical clinical procedures, interpersonal communication, and philosophy, as well as mastering the relevant behavioural skills.

At the highest level of integration, medical officers (senior government officials who are put in charge of medical services in order to advise and lead teams of medical experts in charge of local health systems or hospitals) should integrate completely joint clinical/public health practices. Their responsibility, however, may be thwarted by a debilitating lack of resources (two or three physicians for a population of 200,000 in some rural and suburban Sub-Saharan Africa, for instance).

### Consequences for health knowledge

Medical professionalism and specifically joint clinical/public health practice have implications for medical knowledge, health management, research, and medical education:

- The design and implementation of public health interventions should build on locally available medical knowledge and mores, for example, to adapt disease control guidelines to local conditions.
- Both public health doctors and clinicians should continuously and critically read professional literature respectively drawn from clinical medicine and public health. Accessing and using relevant knowledge from other fields of activity is an often neglected challenge.
- Although eco-biopsychosocial care ideally entails inter-professional teams, some personal, internalised integration of public health and clinical knowledge is required because GPs and family doctors must combine curative and preventive care (see above); tailor this mix to each patient; and *negotiate* therapy, life -style advice, and the use of medical services with the patient (the person-centred care standard).
- If GPs and specialists are expected to set quality of care criteria and therapeutic objectives together (a must when the patient is hospitalised), health systems also need to manage GPs and specialists’ knowledge in such a way that lets the two groups communicate effectively.
- Academia should be in a position to attract physicians capable of conceptualising and transmitting their experience. However, academic circles are increasingly off limits to clinical and public health practitioners because experience and professionalism, as opposed to bibliometrics, are not easily evaluated, and academic salaries may present opportunity costs to physicians.

### Joint clinical / public health medical practice, a professional endeavour

According to Barondess, [20] ‘Professions are complex social structures derived from the guild system of specialised competences intended to organise specialised and complex bodies of knowledge in such a way as to address both individual and societal needs. These are the basis of a social contract enfranchising the members of a profession. It makes professional knowledge central to the well-being of today’s society.’

Integrated clinical/public health knowledge is professional in principle because joint clinical/public health practice is a professional endeavour. Indeed, medical practice has evaded standardisation in several domains, as evidenced by the following examples:

- Clinical decisions [21] - because evidence-based medicine has been strained through misappropriation by vested interests, inflexible rules, technology-driven initiatives, and an unmanageable volume of information [22];
- Eco-biopsychosocial care delivery [23, 24] and professional education – because both build upon emotional intelligence (the ability to understand your emotions and those of other people and to behave appropriately in different situations) [25] and communication skills;
- The treatment of multi-pathologies and a goal-oriented clinical approach [26] – since this entails doctor patient negotiation;
- Surgery, radiotherapy and gynaecology – because they require manual skills;
- The physician’s commitment to community health [27] – because public health programmes should be negotiated with at-risk patients in the same way that clinical options should be discussed between the doctor and the patient.
- The ethical nature of clinical decisions – because the motivation that drives medical professionals is intangible [28, 29].

Clinical and public health doctors both need adequate professional autonomy because the quality of care depends on the physician’s professionalism. Similarly, physicians in managerial positions need sufficient autonomy to promote professionalism and ethics in health care services.

### Knowledge gaps in joint clinical and public health practice

Historically, clinical practice and science benefited from the public health science which has delivered a large body of concepts and quantitative inputs. To name just a few such inputs,

- Clinical epidemiology and evidence-based medicine (EBM) provided treatment guidelines;
- Epidemiology informed the design of prevention and health promotion interventions in curative care practice;
- The surveillance of nosocomial infections shaped antibiotic therapy in hospitals;
- Pharmaceutical evaluations built upon and enlarged (clinical) epidemiology methodology; and
- Health management science addressed medical communication and coordination.

However, public health science could have served care much better if it had not produced exclusively *normative* knowledge, knowledge geared towards decision-making, principally (only?) when public health and clinical epidemiology norms and recommendations were grounded in quantitative, probabilistic research. Indeed, in general it has only contributed to doctors’ and managers’ decision-making when the underlying rationale was quantifiable, for example, based on morbidity and mortality statistics; on the predictive value of signs, symptoms, and test results; or on the effectiveness and cost of interventions.

An examination of joint clinical/public health practice reveals knowledge gaps in both fields. Tasche et al. ([30], cited by De Maeseneer, [31]) analysed 70 guidelines issued by the Dutch College of GPs and identified 875 relevant clinical questions with no answer in published work.

In many countries, most physicians lack insights into:

- The management of professional organisations, for example, how to organise teamwork, to improve clinical coordination, and to lead action-research;
- Reflective methods used to improve medical practice and service delivery; and
- Techniques able to (de-)centralise medical technologies away from hospitals into first-line services and also, the reverse, in order to improve accessibility and efficiency of care.

Similarly, public health science has benefited from clinical knowledge when developing disease control interventions. However, the prevailing epistemological discourse offered clinicians only a minor role in public health programmes. Indeed, over the past half a century or so clinical disciplines allied to traditional public health programmes were usually mobilised according to a standard disease control pattern already outlined in 1965: [32].

**Table:** 

• In theory, epidemiologists chose the priority diseases to be controlled. In practice, most of the current 132 international disease-specific programmes said to be ‘Global Health Initiatives’ are the result of commercial imperatives; epidemiological studies rarely entered the picture [33] Notice that access to care for the poor (roughly 70% of African and Indian populations) was largely limited to priority disease-control interventions, with fieldwork usually the responsibility of auxiliary health workers (and, to a lesser extent, first-line nurse practitioners). This increased the separation between public health activities and clinical medicine. • Health economists set the programmes’ structures. Historically, they preferred vertical to horizontal programmes for considerations of efficiency. In practice, these programmes were operationally integrated in health care services but remained administratively autonomous, leading to dysfunctional management and bureaucratic inflation in low- and middle-income countries (LMICs) [8], so further weakening health systems in developing countries [34]. • Physicians and biologists decided the operational interventions to be led by public health programmes. • Operational and implementation research (increasingly involving anthropologists) was established, for example to determine how to deliver these interventions and to improve population compliance [35]. • Finally, programme evaluations were left to economists and epidemiologists.	

The political imperative of this discourse was central to the development of the political economy of care in LMICs because it legitimised limiting public service’ activities to mere disease control on the grounds of cost-efficiency. In so doing, this epistemological discourse often misused allocative efficiency, which was confused with technical efficiency [36].

As a science of disease control, public health generally gave health care management short shrift. It frequently overlooked the often most important single health status determinant, i.e. healthcare. Thus public health specialists frequently neglected;

- The importance of the accessibility to care and its impact on population health (e.g., to improve early detection, care continuity, and yes, to recruit patients for public health interventions).
- The professional expertise required for the application of clinical and disease control guidelines.
- The multi-causality of disease with which clinical practitioners must grapple.
- The need to set conditional priorities within and between public health programmes.

Because these neglected themes are crucial to clinical and public health medicine, we question the very fact of their separation as well as the distinction between scientific and professional knowledge.

### Integrated clinical/public health knowledge

We propose a typology based on a knowledge merger designed to inform medical education, training and research, which is consistent with the universal right to healthcare. Basing a typology of medical knowledge on concepts of cognitive psychology permits professionals to specify how knowledge is to be transmitted, taught, and assessed. This issue of medical knowledge transmission is crucial, as the written word does not lend itself well to improving the status of the patient, and the knowledge and behaviour of practitioners [37]. We have chosen one that is inspired by that of Malglaive and Piaget to define four categories of interdependent knowledge, namely, the skills; the procedural, the practical and the theoretical knowledge [38]. We shall see at the end of this section that these 4 categories are found in the structure of action research (AR), a research methodology particularly suited to the development of professional knowledge.

### Skills

Behavioural skills include communication, emotional intelligence, reflection, conflict resolution, self-organisation, ability to balance work and life, time management, stress management, resilience, and patience. Together with manual skills, they are of primary importance in medicine. Manual skills are, of course, necessary in clinical medicine, but also in the interface with machines, for example in radiotherapy or endoscopic surgery. Skills are especially important in clinical medicine because they concern the clinician’s interaction with an individual and his/her family, However it may be argued that they are important also in public health, as disease control programmes should be negotiated with communities and authorities.

Skill transmission requires demonstrations, observations and technical supervision [39]. Teaching programmes should systematize skill acquisition. The importance of distinguishing skills from other health care knowledge appears to be understood in very few medical schools save some that have developed ‘problem-based learning’ programs: these are more the exception than the rule (Maastricht; Barts, Queen Mary; East Anglia; and Glasgow).

### Procedural knowledge

Procedural knowledge is often defined as knowledge exercised in the performance of a task. We use it in the more restrictive sense of the knowledge required to apply clinical / public health guidelines (or standard operating procedures - SOP). The application of SOP guidelines is especially complex in medicine, because the principles of Evidence Based Medicine (EBM) require that the values underlying a SOP be compared with those of the patient so as to define the therapeutic process to be undertaken – an especially important challenge in multi-pathology, because achieving objectives in treating one disease may be detrimental to therapeutic effectiveness in another.

For medicine to serve the universal right to health care, clinical medicine and public health procedures must be integrated as frequently as possible. For example, general practice, paediatrics and gynaecology include a significant component of preventive medicine whilst preventive medicine in well-baby clinics, prenatal, and geriatric consultations can be partly standardised in order to rule out or treat relevant pathologies (of the new born and the infant, the pregnant woman and the elderly, respectively).

Clinical and public health guidelines should exhibit two critical characteristics that they frequently lack:

1. Certain criteria for care quality can be defined at opposite ends of a notional scale, such as patient safety (with, for example, complaint medicalisation) and autonomy (vis-à-vis the disease and its medical solution), or the effectiveness and efficiency of treatment. If the doctor maximizes the first, (s)he reduces the second, and vice versa. To enable the physician’s to assess these guidelines in individual circumstances, guideline designers should make explicit the balance between the contradictory qualities of care criteria that govern their conception.
2. Guidelines should offer alternative options to physicians to resolve clinical challenges (for example using different referral values) and so permit them to negotiate clinical treatment plans with their patients. Indeed, patients can only sensibly choose their treatment if alternative options and an explanation of their pros and cons are clearly offered. As for lifestyle clinical advice[s], it remains irrelevant if the patient does not have a choice. In practice, clinical guidelines rarely propose options, either because their commercially focused design only considers efficiency, or because their designers did not think of it.

The transmission and improvement of skills and procedural knowledge require educational supervision, intervision (mutual supervision), flow process auditing (to identify the hurdles a patient meets during his journey through the health system), action-research, and other reflective techniques. Medical faculties and health services should systematize the organisation of rotations and internships during medical training because this effects a lifelong improvement of problem-solving capacity of health professionals. In practice, faculties and health services rarely do so.

### Practical knowledge

Malglaive [38] adds a ‘practical knowledge’ category that we apply to neo-Hippocratic medical practice and its management. Practical knowledge is needed to formulate advice[s] and norms for action in defined environments. It thus informs the design of new procedures and their use in new contexts.

To serve the universal right to healthcare, practical knowledge addresses a wide array of interconnected clinical and public health topics, with quality criteria addressing both domains. For example:

- The professionalization of physicians and that of other health proficients (e.g. in family medicine practiced by nurses in sub-Saharan Africa). Quality criteria may include ethical behaviour, problem-solving capacity, material conditions of the medical practice, and self-reflection capacity;
- The improvement of quality of care (for example through adding family therapy and social assistance to general practice);
- The optimisation of the clinical management of syndromes (say, gonorrhoea); of diagnosis and treatment of a given disease (say, the first and second line treatment of tuberculosis); of disease control (e.g.., diabetes or malaria within a defined area);
- Medical, disease prevention amongst high-risk groups (undocumented migrants in Belgium for instance);
- Improvement in access to health care (for example, access to general medicine) or to drugs for both the general population and for patients with special needs (e,g. patients referred to hospital);
- The optimisation of resource utilisation and procurement (drugs, medical equipment, finances, staff);
- The improvement of the health environment for patients and high-risk groups;
- The organisation of health services and systems (to improve, for example, care coordination between GPs and specialists).

The epistemic unit of practical knowledge can be defined as “strategy”, a representation of ways and means to achieve a goal. “Strategies” can serve as an action plan, action hypothesis, advice, standard, or the basis for an assessment. Strategies link endpoint objectives to a complex sequence of analysis, decision, action, and evaluation. To deal with complex realities they simultaneously address a number of resources, various processes, and many outputs – all topics on which the scientific public health literature is limited. Strategies can be described and evaluated and hypotheses about the conditions of their success or failure can be formulated to define their domains of validity. Multidisciplinary models and concepts are the ones that best describe such conditions. For example, the effectiveness of a vaccination program depends on the socio-cultural characteristics of the population (who decides? The father or the mother?); its geographical distribution; the characteristics of health services; the economic resources of families and those of the program; the level of competence of health professionals; etc.

The joint clinical and public health nature of practical medical knowledge appears in the process of integrating disease control in health care services, and in achieving this whilst strengthening, rather than undermining these services. Disease control programs can reduce access to care in the setting in which they are integrated, imposing on them multiple lines of authority; setting ill-defined priorities and increasing opportunity costs. Inadequate budgets, financial overruns and unrealistic costing; tension between health care professionals over income disparity and problems with sustainability are all too frequent characteristics of such programmes.

The essence of practical medical knowledge is professional and not merely scientific, because:

- The practical knowledge acquired by clinicians and public health physicians should reduce any uncertainty in decision-making, improve programme implementation, promote reflectivity (the quality to reflect i.e. redirect back to the source), and enhance the relevance of evaluation.
- To promote the correct use of EBM, [40] end-user physicians should adapt clinical guidelines to local (epidemiological, cultural, economic, medical) conditions [41]. This is the method by which GPs and the EBM Practice Net adapted the Finnish Duodecim guidelines to Belgian conditions.

Unfortunately, in guiding physicians, Health Maintenance Organisations (HMOs) and government administrations have often over-relied on a biased, unnecessarily strict interpretation of ‘scientific’/biomedical EBM. They continue to apply it despite overlooking medical regulation, [42] neglecting multi-morbidity challenges posed by ageing populations [43–45] (guidelines often map poorly with complex multi-morbidity), [22] and disregarding aspects of care that escape standardisation.

As a professional endeavour, the transmission of practical knowledge benefits from exchanges based on the experience of learners, and exchanges between them and their teachers. Scientific representations are often an inappropriate method to transmit this.

### Theoretical knowledge

Theoretical knowledge relates to the laws of existence and the constitution and functioning of reality – i.e., why something is true. Theoretical knowledge teaches reasoning, techniques and theory of knowledge. Below, we examine some of the characteristics of the theories that support medical professionalism.

First, they often rely on interconnected clinical and public health concepts. One can only understand domestic violence, against women, for example, with concepts of sociology (this violence is not explained in the same way in endogamous and exogamous societies because the free sexuality of women threatens the family architecture of endogamous societies much more than that of the others).

Likewise, consider a basic task of a clinician: diagnosis. Clinical epidemiology and epidemiology ought to be taught together, because the positive predictive value of a sign, a symptom or a test result is a function of the disease prevalence expressed as:

$$ \mathrm{PPV}=\left(\mathrm{SS}\ \mathrm{X}\ \mathrm{d}\right)/\Big(\left(\mathrm{SS}\ \mathrm{d}+\left(1-\mathrm{SP}\right)\left(1-\mathrm{d}\right)\right). $$

Where PPV is the positive predictive value of a sign or a test; SS is its sensitivity; SP is its specificity; and d is the disease prevalence [46].

This formula shows that the predictive value of a sign, symptom or test depends on the local prevalence of the disease that it predicts. Therefore, the value of a symptom is not the same in the patient base of GPs compared to specialists. The physicians’ clinical experience is therefore radically different. Nevertheless, in most universities, it is mainly specialists who teach semiology to the future GPs.

Second, professional research methodologies are interdisciplinary, not merely multidisciplinary. Interdisciplinarity is the interaction between disciplines. It leads to the mutual integration of concepts and methodologies while multidisciplinarity is the simultaneous use of sciences belonging to different fields. Interdisciplinarity opens the door to ad-hoc, original study methods and is often a sine-qua-non for the relevance of theoretical work [47] that (in this case) supports the practice of medicine. Unfortunately, universities today undermine interdisciplinarity with a competition linked to research finance; the publication race; reduced advancement prospects suffered by academics who maintain a professional practice; and scientific specialisation as the basis for career strategies.

Third, medical curricula often neglect two disciplines pivotal to understand and support the personal, professional development of physicians: psychology and philosophy. A knowledge of professional psychology is indispensable in understanding physicians’ intangible motivations and professionals’ wellbeing in general [48]. Values that govern (and should govern) medical and public health practice are derived from moral philosophy.

Finally, policy studies and health systems research should pay attention to the political sciences, [49] the political economics of health care, and history, because the construction of national health systems spans generations and history reveals the political, economic and social determinants of health structures, medical cultures and praxis. In practice, health policy studies rarely set outcomes in context as reputable journalist would.

This typology of medical knowledge closely overlaps the stages of action research:

- The design of a strategy (for example, to control AIDS) requires practical knowledge. A sound strategy is a prerequisite to develop procedures, such as the conduct of a follow-up consultation.
- Procedures are always implemented relying on the doctor’s procedural knowledge and know-how.
- The strategy design depends on the problem it intends to solve, on prior (practical and theoretical) knowledge and on a model (that represents the characteristics of the environment, the problem, the strategy and its expected effect).
- Finally, the strategy evaluation assumes the assessment of its design and of the procedures implementation.

Even though medical interventions benefit from science, they are, in essence, professional: science cannot standardise eco-biopsychosocial decisions; doctor-patient negotiations; emotional intelligence; manual and behavioural skills; and resolution of ethical conflicts.

## Conclusion

The divorce between medical and public health practices results from a history of individual care for the rich and public health interventions for the poor, whether in the nineteenth century in England or in the 20th-21st centuries in Africa. Historically, relations between social classes had negative impacts not only on the health sector’s functions and structures but also on the delineation of scientific fields and of medical epistemology.

Joint clinical/public health practice is needed to improve both access to and quality of care. We set out to demonstrate that knowledge required to support good medical practice revolves around reconciling clinical and public health science. To do so, we conceived of a neo-Hippocratic medical and health management science, an organised field of knowledge with normative, social, and professional objectives and values. Collectively developed by physicians and health professionals, this integrated medical knowledge would support and reinforce joint clinical/public health practice, help underwrite the right to health care, ensure that the societal concerns of public health are taken into account, improve medical professionalism, and bolster the neo-Hippocratic rationale of medical practice.

Such professional (and scientific) effort assumes that it is necessary to revisit the boundary between clinical and public health medicine, because;

- Medical know-how is predominantly clinical.
- Procedural knowledge addresses clinical, public health and managerial challenges, and succeeds more completely when it addresses them together.
- Practical knowledge refers to these same domains.
- Theoretical knowledge addresses both clinical and public health medicine and introduces the benefits of an interdisciplinary approach.
- Procedural and practical medical knowledge should refer to one identical set of medical values and criteria.

How would this science of medical professionalism, joint clinical/public health practice and policy management distinguish itself from the science of commercial medical practice and of the industrial, generic science of management?

The latter envisages public-facing health practitioners as technicians and employees enjoying little autonomy. By contrast, the former acknowledges that both the quality of care and physicians’ motivation require sufficient professional autonomy within a clear framework, because care incorporates the professionals’ labour and reflects their intangible motivation. The organisational consequences of this autonomy are immense and include such elements as symbolic incentives, evaluations, information systems, design of clinical guidelines, transmission of knowledge, etc.

Second, health care is increasingly quantified because markets tend to restrict payment to what can be measured. That generates huge bureaucratic data needs, and jeopardises the indispensable medical secret [50]. Health care markets have impacted medical and public health sciences because of academia’s involvement in the health care industry. This is perhaps best reflected in the unwarranted utilisation of probabilistic methods that has led to the crisis in EBM exposed by T. Greenhalgh [22] and in public health.

Third, in contrast to what generic management generally permits, medical professionalism and joint clinical and public health practice treat quality of care as a largely unquantifiable parameter because it deals with the importance of personal skills, communication, ethics, reflectivity for the quality of care; and the complexity of biopsychosocial decisions. Neo-Hippocratic medicine and management is able to address professionals’ personal development, philosophical thinking, reflectivity, coordination, and teamwork needed to underwrite the physicians’ autonomy and to encourage their symbolic motivation. Admittedly, quality of care is not an entirely qualitative concept, as joint practices benefit from quantitative indicators, for example to monitor progress made in achieving care quality, patient accessibility, and disease control.

As a political consequence, health systems should

- Individualise continuing medical education as far as possible;
- Stimulate the development of the physicians’ professional culture and self-awareness;
- Give clinically experienced physicians the opportunity to acquire managerial and educational positions, in medical faculties as in schools of public health.
- Promote technical and psychological coaching by other, more experienced doctors.

As an academic consequence, unless medical schools and schools of public health distinguish between the physician’s professional and scientific knowledge and make plans to commission research into the former, they will not have the tools necessary to continue training competent physicians.

Unless clinicians, public health physicians, and health care managers acknowledge the social and professional dimensions of medical practice and the need for dual clinical/public health practice, they will not have the means to uphold the ethical dimension of medical practice and the accessibility of care in a world of gross disparities in the health care resources earmarked for different groups.

## Data Availability

Data sharing is not applicable to this article as no datasets were generated or analysed during the current study.

## Abbreviations

EBM: Evidence-Based Medicine
GP: General Practitioner
HMO: Health Maintenance Organization
HSR: Health Systems Research
LMIC: Low and Middle Income Countries
